# Molecular Characterization of *Giardia duodenalis* in Children and Adults Sampled in Algeria

**DOI:** 10.3390/microorganisms9010054

**Published:** 2020-12-28

**Authors:** Salem Belkessa, Daniel Thomas-Lopez, Karim Houali, Farida Ghalmi, Christen Rune Stensvold

**Affiliations:** 1 Laboratory of Analytical Biochemistry and Biotechnology (LABAB), Department of Biochemistry and Microbiology, Faculty of Biological and Agronomic Sciences, Mouloud Mammeri University of Tizi Ouzou, Tizi Ouzou 15000, Algeria; salembelkessa@yahoo.com (S.B.); houalitizi@yahoo.fr (K.H.); 2 Department of Natural and Life Sciences, Faculty of Exact Sciences and Natural and Life Sciences, Mohamed Khider University of Biskra, Biskra 07000, Algeria; 3 Laboratory of Parasitology, Department of Bacteria, Parasites & Fungi, Infectious Disease Preparedness, Statens Serum Institut, Artillerivej 5, DK-2300 Copenhagen S, Denmark; datl@ssi.dk; 4 European Public Health Microbiology Training Programme (EUPHEM), European Centre for Disease Prevention and Control (ECDC), 16973 Solna, Sweden; 5 Higher National Veterinary School of Algiers, El Alia, Algiers 16000, Algeria; fghalmi@yahoo.fr

**Keywords:** allelic sequence heterozygosity (ASH), diarrhea, *Giardia duodenalis*, molecular epidemiology, parasite, real-time PCR

## Abstract

The molecular epidemiology of giardiasis in Africa remains unclear. A study was carried out across four hospitals in Algeria. A total of 119 fecal samples from 55 children, 37 adults, and 27 individuals of undetermined age, all scored positive for intestinal parasites by microscopy, and were screened by real-time PCR for *Giardia*. Molecular characterization of *Giardia* was performed by assemblage-specific PCR and PCR targeting the triose phosphate isomerase gene (*tpi*). Of the 119 samples, 80 (67%) were *Giardia*-positive by real-time PCR. For 48 moderately-highly real-time PCR-positive samples, *tpi* genotyping assigned 22 samples to Assemblage A and 26 to Assemblage B. Contrary to Assemblage A, Assemblage B exhibited substantial genetic diversity and allelic heterozygosity. Assemblage-specific PCR proved to be specific for discriminating Assemblage A or B but not as sensitive as *tpi* genotyping. We confirmed that real-time PCR is more sensitive than microscopy for detecting *Giardia* in stool samples and that robust amplification and sequencing of the *tpi* gene is feasible when moderate-to-strongly real-time PCR-positive samples are used. This study is one of the few performed in Africa providing genotyping data on *Giardia* infections in humans. Both assemblages A and B were commonly seen and not associated with specific sociodemographic data.

## 1. Introduction

Enteric parasites are significant contributors to global diarrheal disease and other intestinal symptoms [1] and, among these, *Giardia duodenalis* (syn. *Giardia lamblia, Giardia intestinalis*) is a flagellated zoonotic parasite commonly found in the intestinal tract of humans and animals, resulting in large numbers of gastrointestinal infections [2]. Worldwide, up to 280 million people are estimated to be infected with *G. duodenalis* [2,3,4,5]. The reported prevalence of *Giardia* infections in humans ranges between 0.4% and 7.5% in developed countries, and between 8% and 30% in developing countries [2]. Symptoms vary in intensity, which may be attributable to both host and parasite factors [6]; infection is considered asymptomatic in approximately 50% of the cases, while severe diarrhea combined with vomiting, bloating, nausea, and/or fatigue can be seen in symptomatic patients, and impaired growth may be seen in children [5].

Owing to its extensive genetic variation [7,8], *G. duodenalis* is considered a species complex, involving eight genetically distinct groups or genotypes (assemblages A–H) with varying zoonotic potential and host specificity [9]. Assemblage B in particular exhibits allelic sequence heterozygosity (ASH) across multiple loci [10]. Assemblages can be identified by polymerase chain reaction (PCR), either in combination with restriction fragment length polymorphism (RFLP) or sequencing of the PCR amplicons [11,12,13]. Because of differences in the sensitivity of the PCR assays and occasional discrepant genotyping results among genetic loci, it has been recommended to determine *G. duodenalis* assemblage by multilocus analysis, which provides higher resolution [14] and involves sequencing of the small subunit (SSU) of the nuclear ribosomal RNA (18S rRNA gene), β-giardin (*bg*), glutamate dehydrogenase (*gdh*), and/or triose phosphate isomerase (*tpi*) genes [2,4,15,16]. The *tpi* gene has generally proved to be a robust marker and may be used alone to provide baseline information where no a priori knowledge is available [17]. Another method was developed in 2012 to detect and differentiate assemblages A and B in human fecal samples [18]. The method involves a single-step PCR using assemblage-specific primers and relies on the differences in PCR product sizes for assemblages A and B visualized by gel electrophoresis. This method has proven robust for detecting mixed infections and in terms of applicability in laboratories with basic molecular equipment [18]. In Europe and America, the epidemiology of *Giardia* human infections is relatively well documented, even at the assemblage level. However, in Africa, very little data is available [19], and the extent of ASH in *G. duodenalis* in humans in Africa has been elucidated only to a very limited extent [20]. Particularly in Algeria, there is—to our knowledge—only one *Giardia* genotyping study on human isolates; however, no DNA sequencing data were available in that study [21]. Therefore, in order to provide some baseline data on the molecular epidemiology of *Giardia* infections in children and adults in Algeria, we carried out a study to characterize *G. duodenalis* from two separate geographical areas, using real-time PCR, assemblage-specific PCR, *tpi*-sequencing-based genotyping, and analysis of ASH within *Giardia* assemblages.

## 2. Materials and Methods

### 2.1. Stool Sample Collection and Participants’ Demographic Characteristics

The study was carried out from 2013 to 2018 across two hospitals in the Algiers metropolitan area (Centre Hospitalo-Universitaire Issad Hassani, Beni Messous, and Centre Hospitalo-Universitaire Nafissa Hamoud [ex-Hôpital Parnet], Hussein Dey) and two hospitals in the region of Biskra (Tolga Hospital and Doctor Saadane Hospital).

A total of 119 fecal samples collected from 119 individuals suspected of parasitosis and/or suffering from gastrointestinal symptoms (i.e., diarrhea, vomiting, and/or abdominal cramps) and scored as parasite-positive by direct wet mount microscopy with formalin-ether concentration technique for ova and parasites, were included in our study. Forty-eight individuals were females, 66 were males, and for five individuals, information on sex was not available. Fifty-five patients were younger than 15 years and 37 were older (age range, 2–74 years [median, 8 years; interquartile range (IQR), 4.25–24]); for 27 patients, the age was unknown. Study individuals below the age of 15 were classified as children according to similar studies [22,23,24].

All samples were screened by real–time PCR for *Giardia* (see below). Some of the samples (*n* = 22) used in the present study were also used in our recent study describing the prevalence of *Giardia* and various parasites in Algerian children and adults [25] (manuscript accepted for publication).

### 2.2. Genomic DNA Extraction and Purification

All stool samples had been stored in either potassium dichromate (2.5%) or ethanol (70%) upon collection (of note, to ensure better preservation, approximately one-third of stool samples were supplemented by two-thirds of preserving agent in dry tubes). To remove the preserving agent, samples were washed in PBS once prior to DNA extraction. Total DNA was extracted from stool samples using the NucliSENS^®^ easyMAG^®^ system (bioMérieux, Marcy-l’Étoile, France) following the manufacturer’s instructions with some modifications [26,27]. Briefly, approximately 200 mg of each sample were placed in a 2 mL microtube, mixed with 400 μL Lysis Buffer, and vortexed using Mylab (Vortex-Mixer SLV-6, Seoulin Bioscience Co., Ltd., Seoul, Korea) for 2 min to ensure thorough fragmentation and homogenization. The samples were subsequently centrifuged for 5 min at 16,000 rpm. From each sample, 100 µL of supernatant were lysed in the NucliSENS^®^ easyMAG^®^ apparatus. Subsequently, 60 μL of magnetic silica were added to each sample and thoroughly mixed. The extracted DNA samples were afterward stored at −20 °C until use.

### 2.3. Real-Time PCR

All study samples (*N* = 119) were screened for *Giardia* by in-house real-time PCR, in place at Statens Serum Institut, which amplifies a 62-bp region of the small-subunit ribosomal RNA (SSU rRNA) gene of *Giardia*, using primers and probes previously published [28] (Table 1).

The 25-µL real-time PCR assay mixture consisted of 0.2 µL IMMOLASE™ DNA Polymerase (Bioline), 5 µL 10× ImmoBuffer, 1.25 µL (1 µM) of the primers Giardia-80F and Giardia-127R, 0.125 µL (0.075 µM) of the probe (Giardia 105T), and 5 µL of DNA eluate. The real-time PCR was carried out using an Applied Biosystems 7500 Fast Real-Time PCR Thermocycler (Thermo Fisher Scientific) with the following cycling conditions: initial denaturation at 95 °C for 10 min, followed by 50 cycles of 95 °C for 15 s, and 60 °C for 60 s. PCR products were analyzed using Sequence Detection Software v.2.3 (Thermo Fisher Scientific). A sample was considered positive if an exponential curve was observed with a Ct-value ≤ 42. Negative (water) and positive controls (DNA for *G. duodenalis*) were included in each run as well as inhibition controls.

### 2.4. Molecular Typing

Assemblage-specific PCR (4E1-HP method [18]) was performed on a 96-well LifeECO Thermocycler (Bioer Technology) using the Platinum^®^ Taq DNA Polymerase Master Mix (Invitrogen). The final concentration of each primer used in PCR reactions was 0.4 pM, and the sequences of each primer pair are displayed in Table 1. All reactions followed the same thermocycler programming: an activation-denaturation step at 94 °C for 5 min followed by 40 cycles, each consisting of 94 °C for 30 s, an annealing step of 30 s at 56 °C, and an extension step of 72 °C for 30 s. A final extension step was performed at 72 °C for 7 min. PCR products were analyzed by electrophoresis (90 V, 60 min) on TBE 1x 1.5% agarose gels stained with EZ-Vision^®^ DNA dye (Amresco Inc., Solon, OH, USA) and photographed with BioDoc-it™ imaging system (2UV Transilluminator). A 1 kb DNA molecular weight marker GeneRuler DNA Ladder Mix (Thermo Scientific™, Waltham, MA, USA) was used. Assemblages were identified based on the size of the PCR products relative to the positive control DNAs (Assemblage A and Assemblage B) kindly provided by the European Union Reference Laboratory for Parasites, Istituto Superiore di Sanità, Rome, Italy. The sample was considered positive for *G. duodenalis* Assemblage A or Assemblage B if we observed a 165-bp or a 272-bp PCR product, respectively [18]; a sample was considered positive for both assemblages (mixed infection) if both PCR products were present. If a PCR product of a different size was observed, this finding was reported as well (Table S1).

Most *Giardia*-positive samples with a Ct value < 32 DNA were available for typing, and these were genotyped to assemblage level using a nested PCR assay for amplification of a 530-bp fragment of the *tpi* gene [17]; this Ct value threshold was chosen based on the previous experience that samples with higher Ct values were unlikely to yield *tpi* sequences of sufficient quality. We therefore considered samples with Ct values < 32 as moderate-to-strongly *Giardia-*positive samples.

The PCR assays were carried out in total volumes of 25 μL, consisting of 12.5 μL of ReadyMix™ Taq PCR Reaction Mix (Sigma-Aldrich Co. LLC, CA, USA) (PCR Buffer, MgCl2, dNTP, DNA Polymerase Taq polymerase), 1 μL (0.4 µM) of each of external primers AL3543 and AL3546, 8.5 μL of Invitrogen™ UltraPure™ DNase/RNase-Free Distilled Water (Thermo Fisher Scientific, Carlsbad, CA, USA), and 2 μL of DNA for the primary amplification. PCR was performed with an initial hot start of 94 °C for 5 min, 35 cycles of 94 °C for 45 s, 50 °C for 45 s, and 72 °C for 60 s, followed by a final extension of 72 °C for 10 min using a 96-well LifeECO Thermocycler. The same conditions were applied to the second PCR reaction where 2 μL of PCR product from the primary reaction and 1 μL (0.4 µM) of the internal primers AL3544 and AL3545 were used for the reaction [17]. PCR-positive samples were purified with QIAquick^®^ PCR Purification Kit (Qiagen, Hilden, Germany) according to the manufacturer’s instructions, and sequenced bi-directionally by Eurofins MWG Operon (Ebersberg, Germany).

### 2.5. Sequence Analysis

DNA sequences specific to the *tpi* gene were edited and trimmed using Staden Package 2.0.0b11-2016 software and aligned using online tools (http://multalin.toulouse.inra.fr/). Analysis of ASH in the *tpi* sequences involved the identification of ‘double peaks’; i.e., overlapping nucleotides. *tpi* sequences were translated into amino acid (aa) sequences using an online translation tool (http://web.expasy.org/translate) and aligned to identify differences in aa sequences reflecting any double peaks. Reference sequences from the major *G. duodenalis* assemblages and subtypes used for comparison were chosen based on previous studies [10,22,29,30,31] and retrieved from GenBank (accession numbers: AF069556, AF069557, MF169203, DQ650648, MK509037, MH644770, JX266842, AY228628, AF069560, AY228641, DQ246216, KF891311, and AF069558). DNA sequences reflecting the *tpi* gene obtained in this study were submitted to GenBank under the accession numbers: MW251133-MW251180.

### 2.6. Statistical Analysis

Real-time PCR cycle threshold (Ct) values were determined using the software StepOne, version 2.3 (Applied Biosystems, Foster City, CA, USA).

Differences in the prevalence of *Giardia* between groups were tested by calculating and comparing Wilson’s confidence intervals (CI) for proportions, supplemented by the N-1 Chi-squared test (https://www.medcalc.org/calc/comparison_of_proportions.php, access date: 10/07/2020).

Differences in Ct values between groups of samples were evaluated using the unpaired *t*-test implemented in Microsoft Excel 2016. The sensitivity and specificity of both microscopy and real-time PCR, using each other as the gold standard, were calculated using an online diagnostic test evaluation calculator (https://www.medcalc.org/calc/diagnostic_test.php). The inter-test agreement between microscopy and real-time PCR was quantified using Cohen’s Kappa index (https://www.graphpad.com/quickcalcs/kappa1/). Differences between the sensitivity and specificity of microscopy and real-time PCR were tested using McNemar test on paired proportions (https://www.scistat.com/statisticaltests/mcnemar.php). Probability (*p*) values < 0.05 were considered to indicate statistical significance.

## 3. Results

### 3.1. Confirmation of Microscopy Results by Giardia-Specific Real-Time PCR

**Figure 1:**
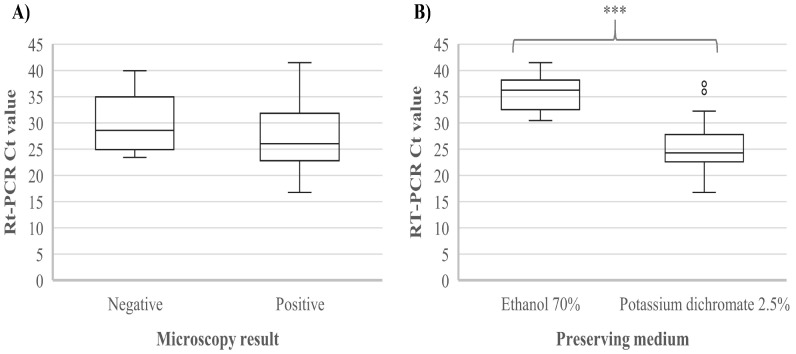
Real-time PCR cycle threshold values according to (**A**) microscopy results (negative vs. positive for *Giardia duodenalis*) and (**B**) preserving medium (ethanol 70% vs. potassium dichromate 2.5%). *** indicates *p* < 0.001.

For this study, we used DNAs from parasite-positive stool samples collected in Algerian hospitals. Of the 119 samples, 80 (67%) were real-time PCR-positive for *Giardia* (median Ct value, 26.56; IQR, 23.11–32.22), of which 66 had been scored as *Giardia*-positive by microscopy (median Ct value for microscopy-positive samples, 26.04; IQR, 22.78–31.86). Fourteen samples that were positive by real-time PCR had been scored negative for *Giardia* by microscopy (median Ct value, 28.59; IQR, 24.92–34.95). Ct values of samples positive by both real-time PCR and microscopy were lower than the Ct values of samples positive by real-time PCR but negative by microscopy, but not significantly so (*p* = 0.13; Figure 1A). The remaining 39 (33%) samples were real-time PCR-negative for *Giardia*; among these, seven had been scored positive for *Giardia* by microscopy, and the remaining 32 were negative by both real-time PCR and microscopy.

As assessed by real-time PCR, *Giardia* was significantly more common in children, with 45/55 (81.8% [CI, 69.7–89.8%]) positive children compared with 11/37 (29.7% [CI, 17.5–45.8%]) positive adults (*p* < 0.001).

The Ct values of the samples that had been stored in potassium dichromate (2.5%) were significantly lower than the Ct values of the samples that had been stored in ethanol (70%) (*p* < 0.001) (Figure 1B). As most individuals of known age were children, we performed the same analysis excluding the adults and individuals of unknown age to eliminate any potential effect of age, and similar results were obtained (*p* < 0.001) (data not shown). The sensitivity of real-time PCR and microscopy using each other as the gold standard was 90.4% and 82.5%, while the specificity was 69.6% and 82%, respectively. Cohen’s Kappa value for real-time PCR and microscopy was 0.61, indicating substantial agreement.

### 3.2. Assemblage-Specific Analysis

Out of the 80 *Giardia*-positive samples by real-time PCR, 4 (5%) and 21 (26%) samples were scored as Assemblage A and Assemblage B, respectively, using assemblage-specific PCR. The remaining 55 (69%) *Giardia*-positive samples failed to produce a clear result by gel analysis; among these, 8 (10%) samples yielded extra/multiple PCR products, including the *Giardia* Assemblage B-specific product; the other 47 (59%) samples exhibited no visible PCR products. The median Ct value for assemblage-specific PCR-positive samples was significantly lower (23.91; IQR, 22.75–26.00) than the median Ct value for assemblage-specific PCR-negative samples (30.98; IQR, 24.24–36.68) (*p* < 0.001).

In order to validate the results obtained with assemblage-specific PCR assay and obtain sequence information about the samples with no PCR products visible by gel analysis, we performed *tpi* genotyping. For the 48 (61%) samples that had Ct values lower than 32 (median Ct value, 24.07; IQR, 22.61–26.63) and for which DNA was available for typing, the *tpi* gene was successfully amplified by nested PCR and sequenced (of note, three samples analyzed with the assemblage-specific assay were not submitted to *tpi* genotyping due to lack of DNA availability). Out of the 48 *tpi* sequences, sequence analysis resulted in 22 (46%) Assemblage A sequences belonging to samples from 10 children, 6 adults, and 6 individuals of unknown age. The remaining 26 (54%) sequences were belonged to Assemblage B and corresponded to samples from 15 children, one adult, and 10 of unknown age (Table 2; Table S1). The samples belonging to Assemblage A were all 100% identical to the A2 subtype reference sequence AF069557 available in GenBank. Meanwhile, the samples pertaining to Assemblage B could not be assigned to the sub-assemblage level by alignment, and therefore they are referred to as Assemblage B only.

### 3.3. Allelic Sequence Heterozygosity (ASH)

For the 22 *Giardia* Assemblage A samples, all 22 sequences were identical, and sequence analysis of the *tpi* gene revealed unambiguous sequence data with no evidence of ASH.

For the 26 Assemblage B samples, however, sequencing of *tpi* PCR products produced no less than 21 distinct sequence variants (Table S2). Seven sequences had no visible double peak at any nucleotide position. Of these sequences, four (ALG-007, ALG-0017, ALG-0019, and ALG-0040) were 100% identical to the BIV reference sequence Ad-19 (GenBank accession no. AF069560) (Table S2). The 19 remaining Assemblage B sequences had overlapping nucleotides (double peaks) in at least one position. Among these sequences, ALG-0022, ALG-0025, and ALG-0031 sequences were 100% identical, with double peaks at the following positions: 77, 162, 165, and 168.

The translation of nucleotide sequences into aa sequences revealed both silent and non-synonymous mutations among the *Giardia* Assemblage B sequences. All observed substitution patterns were transition mutations (T↔C or A↔G), except for one transversion mutation (A↔T) observed in the sample ALG-0044. The nucleotide substitutions of seven sequences (ALG-0026, ALG-0030, ALG-0035, ALG-0038, ALG-0041, ALG-0048, and ALG-0051) corresponded to silent mutations and so did not cause any change in the aa sequence, while the nucleotides substitutions observed in 12 sequences (ALG-003, ALG-008, ALG-0020, ALG-0022, ALG-0023, ALG-0025, ALG-0031, ALG-0032, ALG-0037, ALG-0043, ALG-0044, and ALG-0047) were non-synonymous mutations (Table S3).

## 4. Discussion

In this study, we have provided some baseline information on the *Giardia* assemblages circulating in the human population in Algeria using sequence-based genotyping, and we provide some of the first genotyping data to emerge in this field from the entire Africa. Moreover, our study provides information on the prevalence of *Giardia* among individuals positive for intestinal parasites in selected areas in Algeria and information on upstream factors potentially influencing the process leading to successful genotyping.

Based on real-time PCR, the overall rate of *Giardia duodenalis* was 67%, with children being more commonly infected than adults (82% vs. 30%), confirming the trend identified in our previous study [25]. Real-time PCR was more sensitive for detecting *Giardia* than microscopy, which is in agreement with previous findings [32,33,34,35]. However, we identified seven samples negative by real-time PCR that were microscopy-positive for *Giardia*. This result might be explained in part by a misinterpretation during diagnosis or by the presence of ‘empty’ cysts, which would be observable by microscopy but would lack genetic material that could be amplified by real-time PCR as previously observed by colleagues [36].

The difference in the Ct values observed when using potassium dichromate (2.5%) or ethanol (70%) for sample storage might indicate that the former is more optimal for the preservation of samples collected for detection and molecular characterization of *Giardia*. This finding should be interpreted with caution, however, since clinical or epidemiological information that could otherwise explain this difference (e.g., association between visitation of samples to preserving agent and clinical severity of infection) was not available for this analysis. Meanwhile, we noticed that DNA extracted from samples collected and stored in potassium dichromate back in 2013 was of sufficient quality for DNA extraction and DNA analysis in 2019 when our laboratory work was carried out, exemplifying the applicability of potassium dichromate for this purpose.

The assemblage-specific PCR assay has proved reliable and sensitive in cases of strongly unbalanced samples with variable proportions of assemblages A and B DNA as well as when extremely diluted samples are used [18]. However, in our study only strongly real-time PCR positive samples gave interpretable results. Meanwhile, assemblage-typing using *tpi* gene as the genetic marker revealed consistent amplification when PCR-positive samples with Ct values ≤ 32 were used. A total of 40% (19/48) samples negative by assemblage-specific PCR were positive by *tpi* typing (Table S1). Noticeably, while most samples identified as Assemblage B-positive by *tpi* sequencing were correctly identified as Assemblage B by assemblage-specific PCR, only four of the 22 samples identified as Assemblage A by *tpi* sequencing were scored as Assemblage A-positive by assemblage-specific PCR; most of the remaining samples were negative. This indicates that the assemblage-specific PCR was specific and that the sensitivity was similar to that of the *tpi* assay for Assemblage B but remarkably less sensitive with regard to Assemblage A.

Overall, Assemblage B was primarily identified in children; however, the majority of *tpi* sequences obtained from individuals of unknown age were also attributed to Assemblage B; hence, we could not with certainty link Assemblage B to young age (Table 2; Table S1).

Extensive genetic variation was observed within *Giardia* Assemblage B sequences, with 21 examples of ASH identified among a total of 19 *Giardia* Assemblage B samples, whereas no genetic variation was noticed within *Giardia* Assemblage A sequences. It has previously been established that the *tpi* gene is highly conserved within Assemblage A [10]. Meanwhile, *tpi* sequencing revealed large genetic variation within *Giardia* Assemblage B samples. The observation of sequence heterogeneity in 19/26 sequences as evidenced by the presence of one or more double peaks in both sequence strands indicates that ASH in Assemblage B is extensive in this population. Although this degree of heterozygosity may be due to mixed infections with parasites of multiple Assemblage B sub-assemblages, it may more likely be due to ASH of nuclei within single cysts as suggested in multiple studies [10,14,20,37,38]. Several studies of diverse hosts have reported that ASH can occur at specific positions, and its configuration may differ from one study to another, which may be due to substantial intragenic variability within Assemblages B sequences. The substitution patterns at positions 91, 162, 165, 168, and 280 have been reported from human samples in Sweden [10], Peru, India, and USA [17,39]. The substitution patterns at positions 216, 297, and 429 have been reported from water samples in USA [39]; additionally, the first (position 216) has been also reported from muskrat in USA [17,39], the second (position 297) from human in Peru [39], and the last (position 429) has been reported from rabbit and human in China and Australia, respectively [39]. The substitution pattern at position 77 has been observed in human samples in central Ethiopia [40] as well as in dog samples in USA [17]. The substitution pattern at position 333 has been reported from dog samples in Germany [39]. In the present study, we took into account only sequencing chromatograms with unambiguous peaks. Double peaks of approximately the same intensity were observed on both forward and reverse DNA strands. Thus, the possibility that low-quality sequencing might be the reason of the high number of allele variants could be ruled out, also because the sequences of the Assemblage A samples presented no background noise. In our study, we observed ASH in 73% of Assemblage B samples.

Our study has certain limitations to consider. First, the study was carried out in only two geographical areas, and we only investigated individuals who had been in contact with the health care system, so the data might not reflect the epidemiological situation in the entire country and in the background population. Additionally, if clinical information had been available to us, we could have investigated whether symptoms or intensity of infection as measured by real-time PCR differed between individuals with Assemblage A and those with Assemblage B infection. Similarly, age was not known for several individuals. If the patients of unknown age were all adults, then the difference in *Giardia* prevalence would not be nearly as large as the one accounted for here. Furthermore, we used only two typing methods (assemblage-specific PCR and *tpi* genotyping) for the assignment of *Giardia* samples at the assemblage level, which is not always reliable knowing that methods using different genetic markers can give different results, and more consistent results are obtained when combining the information from multiple markers [37]. Based on the data at hand, the sub-assemblage calling was not possible due to the genetic diversity and the high substitutions rate of Assemblage B samples [41], and lack of information in the GenBank database. Several previous studies have also reported the difficulty in assigning sub-assemblages in Assemblage B samples [14,42,43,44].

Conclusively, in this study population, most of the parasite-positive children had *Giardia*. We moreover observed that real-time PCR appears to be more sensitive than microscopy for detecting *Giardia* in stool specimens, and robust amplification and sequencing of the *tpi* gene is feasible when samples that are moderate-to-strongly positive real-time PCR are used. We were able to identify that potassium dichromate appears to be a better preserving agent for long-term storage of stool samples collected for *Giardia* detection and differentiation than ethanol. Finally, we observed that most Assemblage B sequences exhibited overlapping nucleotide peaks at specific positions in the chromatograms compared with Assemblage A sequences, suggesting substantial ASH and a clear difference in genetic diversity between assemblages A and B. Complementing to our study, further studies are needed with clear data on age, sex, geographic area, and clinical records including a survey of the background population to identify whether this population is more or less infected than those in touch with the healthcare system and to determine the assemblages in this background population (i.e., in those *Giardia*-infected individuals who do not seek medical treatment/advice), which will inform the future management of *Giardia* infections in Algeria.

## Figures and Tables

**Table 1:** List of oligonucleotides used for real-time PCR, assemblage-specific PCR, and *tpi* gene-based genotyping with indications of the sizes of the amplicons.

Assay	Oligonucleotides (Primer/Probe)	Assemblage	Primer and Probe Sequences (5′-3′)	PCR Product Size (bp)	Reference
Real-time PCR	Giardia-80F Giardia-127R	NA	For GACGGCTCAGGACAACGGTT Rev TTGCCAGCGGTGTCCG	62	[28]
	Probe Giardia-105T	NA	FAM-CCCGCGGCGGTCCCTGCTAG-BHQ-1		
Assemblage-specific PCR	4E1-HP	A B	For AAAGAGATAGTTCGCGATGTC Rev ATTAACAAACAGGGAGACGTATG For GAAGTCATCTCTGGGGCAAG Rev GAAGTCTAGATAAACGTGTCGG	165 272	[18]
*tpi* gene analysis	Giardia AL3543 Giardia AL3546 Giardia AL3544 Giardia AL3545	All assemblages	For AAATIATGCCTGCTCGTCG Rev CAAACCTTITCCGCAAACC For CCCTTCATCGGIGGTAACTT Rev GTGGCCACCACICCCGTGCC	605 530	[17]

NA, not applicable; For, Forward; Rev, Reverse.

**Table 2:** Summary of *Giardia* assemblages detected by *tpi* gene analysis according to the basic demographic characteristics of the study population.

Study Population (*N* = 119)	*Giardia*-Positive (Real-Time PCR)	Assemblage A Positive/Typed Samples ^1^	Assemblage B Positive/Typed Samples ^1^
**Children (*N* = 55)**	**45/55 (82%)**	**10/25 (40%)**	**15/25 (60%)**
Boys (*n* = 30)	24/30 (80%)	5/14 (36%)	9/14 (64%)
Girls (*n* = 25)	21/25 (84%)	5/11 (45%)	6/11 (55%)
**Adults (*N* = 37)**	**11/37 (30%)**	**6/7 (86%)**	**1/7 (14%)**
Males (*n* = 23)	5/23 (22%)	3/3 (100%)	0/3 (00%)
Females (*n* = 14)	6/14 (43%)	3/4 (75%)	1/4 (25%)
**Undetermined age (*N* = 27)**	**24/27 (89%)**	**6/16 (37%)**	**10/16 (63%)**
Males (*n* = 13)	12/13 (92%)	4/8 (50%)	4/8 (50%)
Females (*n* = 9)	9/9 (100%)	1/6 (17%)	5/6 (83%)
Unknown (*n* = 5)	3/5 (60%)	1/2 (50%)	1/2 (50%)
**TOTAL**	**80/119 (67%)**	**22/48 (46%)**	**26/48 (54%)**

^1^ Results of the positive samples referred to assemblage A and B were based on *tpi* gene analysis.

## Data Availability

The data presented in this study are available on request from the corresponding author.

## Supplementary Materials

The following are available online at https://www.mdpi.com/2076-2607/9/1/54/s1, Table S1: Summary of the results obtained using real-time PCR, assemblage-specific PCR, and *tpi* gene analysis of *Giardia*-positive samples collected from two separate geographical areas in Algeria, previously tested positive for intestinal parasites by microscopy of fecal concentrates and preserved in either potassium dichromate 2.5% or ethanol 70% prior to analysis; Table S2: Comparative analysis of the extent of allelic sequence heterozygosity in the *tpi* gene of *Giardia duodenalis* Assemblage B identified in the study; Table S3: Comparative sequence analysis of expressed amino acid substitutions in the *tpi* gene of *Giardia duodenalis* Assemblage B samples identified in the study. Only samples with double peaks resulting in the existence of non-synonymous substitutions were included.
