# Supplementary material for: Molecular Characterization of Giardia duodenalis in Children and Adults Sampled in Algeria
Source: Microorganisms. 2020 Dec 28;9(1):54. doi: 10.3390/microorganisms9010054 (PMC7823855; doi:10.3390/microorganisms9010054)
Supplement: Supplementary file 1 [file microorganisms-09-00054-s001.pdf]

Table S1. Summary of the results obtained using real-time PCR, assemblage-specific PCR, and *tpi* gene analysis of *Giardia*-positive samples collected from two separate geographical areas in Algeria, previously tested positive for intestinal parasites by microscopy of fecal concentrates and preserved in either potassium dichromate 2.5% or ethanol 70% prior to analysis.

Sample ID	Date of sampling	Location	Preserving medium	Sex	Age group	Microscopy for *Giardia duodenalis*	Real-time PCR results (Ct value)	Assemblage-specific PCR result	*tpi* result
**ALG-001**	25/03/2018	Doctor Saadane Hospital - Biskra	Potassium dichromate 2.5%	Female	Child	Positive	16.75	Assemblage B + other band(s)	Assemblage A
**ALG-002**	05/03/2018	Doctor Saadane Hospital - Biskra	Potassium dichromate 2.5%	Female	Adult	Positive	19.27	Negative	Assemblage A
**ALG-003**	03/09/2013	CHU Issad Hassani, Beni Messous - Algiers	Potassium dichromate 2.5%	Female	Undetermined	Positive	19.87	Assemblage B	Assemblage B
**ALG-004**	01/02/2016	CHU Issad Hassani, Beni Messous - Algiers	Potassium dichromate 2.5%	Male	Undetermined	Positive	20.16	Assemblage A	Assemblage A
**ALG-005**	14/11/2018	CHU Issad Hassani, Beni Messous - Algiers	Potassium dichromate 2.5%	Male	Child	Positive	20.93	Negative	Assemblage A
**ALG-006**	19/01/2017	CHU Issad Hassani, Beni Messous - Algiers	Potassium dichromate 2.5%	Undetermined	Undetermined	Positive	20.96	Negative	Assemblage A
**ALG-007**	10/09/2017	CHU Issad Hassani, Beni Messous - Algiers	Potassium dichromate 2.5%	Female	Undetermined	Positive	21.1	Negative	Assemblage B
**ALG-008**	27/08/2013	CHU Issad Hassani, Beni Messous - Algiers	Potassium dichromate 2.5%	Female	Undetermined	Positive	21.11	Assemblage B	Assemblage B
**ALG-009**	19/12/2016	CHU Issad Hassani, Beni Messous - Algiers	Potassium dichromate 2.5%	Female	Undetermined	Positive	21.11	Negative	Assemblage A
**ALG-0010**	11/03/2018	Doctor Saadane Hospital - Biskra	Potassium dichromate 2.5%	Female	Adult	Positive	21.55	Negative	Assemblage A
**ALG-0011**	02/04/2013	CHU Issad Hassani, Beni Messous - Algiers	Potassium dichromate 2.5%	Female	Child	Positive	22.16	Assemblage B	Not performed
**ALG-0012**	18/09/2013	CHU Issad Hassani, Beni Messous - Algiers	Potassium dichromate 2.5%	Female	Child	Positive	22.17	Assemblage B	Not performed
**ALG-0013**	28/01/2013	CHU Issad Hassani, Beni Messous - Algiers	Potassium dichromate 2.5%	Male	Undetermined	Positive	22.49	Negative	Assemblage A
**ALG-0014**	08/10/2013	CHU Issad Hassani, Beni Messous - Algiers	Potassium dichromate 2.5%	Male	Adult	Positive	22.54	Assemblage B	Not performed
**ALG-0015**	06/11/2017	CHU Issad Hassani, Beni Messous - Algiers	Potassium dichromate 2.5%	Male	Undetermined	Positive	22.56	Negative	Assemblage A
**ALG-0016**	02/04/2018	Doctor Saadane Hospital - Biskra	Potassium dichromate 2.5%	Male	Child	Positive	22.78	Assemblage B + other band(s)	Assemblage A
**ALG-0017**	31/05/2018	CHU Issad Hassani, Beni Messous - Algiers	Potassium dichromate 2.5%	Male	Child	Positive	22.79	Assemblage B + other band(s)	Assemblage B
**ALG-0018**	19/09/2017	CHU Issad Hassani, Beni Messous - Algiers	Potassium dichromate 2.5%	Male	Child	Positive	22.79	Negative	Assemblage A
**ALG-0019**	07/01/2016	CHU Issad Hassani, Beni Messous - Algiers	Potassium dichromate 2.5%	Female	Undetermined	Positive	22.97	Assemblage B	Assemblage B
**ALG-0020**	03/10/2013	CHU Issad Hassani, Beni Messous - Algiers	Potassium dichromate 2.5%	Male	Undetermined	Positive	23.07	Assemblage B	Assemblage B
**ALG-0021**	25/02/2018	Doctor Saadane Hospital - Biskra	Potassium dichromate 2.5%	Male	Child	Positive	23.25	Assemblage B + other band(s)	Assemblage A
**ALG-0022**	26/03/2018	Tolga Hospital - Biskra	Potassium dichromate 2.5%	Male	Child	Negative	23.42	Assemblage B	Assemblage B
**ALG-0023**	05/03/2018	Doctor Saadane Hospital - Biskra	Potassium dichromate 2.5%	Female	Child	Positive	23.56	Assemblage B	Assemblage B
**ALG-0024**	27/03/2018	Doctor Saadane Hospital - Biskra	Potassium dichromate 2.5%	Female	Child	Positive	23.6	Negative	Not performed
**ALG-0025**	24/03/2018	Tolga Hospital - Biskra	Potassium dichromate 2.5%	Female	Child	Negative	23.61	Assemblage B	Assemblage B
**ALG-0026**	23/04/2013	CHU Issad Hassani, Beni Messous - Algiers	Potassium dichromate 2.5%	Male	Child	Positive	23.71	Assemblage B	Assemblage B
**ALG-0027**	03/12/2017	CHU Issad Hassani, Beni Messous - Algiers	Potassium dichromate 2.5%	Male	Child	Positive	23.8	Negative	Assemblage A
**ALG-0028**	26/02/2018	Doctor Saadane Hospital - Biskra	Potassium dichromate 2.5%	Male	Adult	Positive	23.91	Assemblage A	Assemblage A
**ALG-0029**	13/02/2018	Doctor Saadane Hospital - Biskra	Potassium dichromate 2.5%	Female	Child	Positive	24.24	Negative	Assemblage A
**ALG-0030**	25/01/2016	CHU Issad Hassani, Beni Messous - Algiers	Potassium dichromate 2.5%	Male	Undetermined	Positive	24.26	Assemblage B	Assemblage B
**ALG-0031**	24/03/2018	Tolga Hospital - Biskra	Potassium dichromate 2.5%	Female	Child	Negative	24.32	Assemblage B	Assemblage B
**ALG-0032**	27/04/2018	CHU Issad Hassani, Beni Messous - Algiers	Potassium dichromate 2.5%	Male	Child	Positive	24.81	Assemblage B	Assemblage B
**ALG-0033**	27/03/2013	CHU Issad Hassani, Beni Messous - Algiers	Potassium dichromate 2.5%	Female	Child	Positive	25.02	Negative	Assemblage A
**ALG-0034**	25/03/2018	Tolga Hospital - Biskra	Potassium dichromate 2.5%	Male	Child	Negative	25.12	Assemblage B	Assemblage B
**ALG-0035**	31/03/2013	CHU Issad Hassani, Beni Messous - Algiers	Potassium dichromate 2.5%	Undetermined	Undetermined	Positive	25.5	Assemblage B + other band(s)	Assemblage B
**ALG-0036**	21/08/2013	CHU Issad Hassani, Beni Messous - Algiers	Potassium dichromate 2.5%	Male	Undetermined	Positive	25.5	Assemblage B	Assemblage B
**ALG-0037**	24/03/2018	Tolga Hospital - Biskra	Potassium dichromate 2.5%	Male	Child	Negative	25.59	Assemblage B	Assemblage B
**ALG-0038**	10/04/2018	Tolga Hospital - Biskra	Potassium dichromate 2.5%	Male	Child	Positive	25.84	Negative	Assemblage B
**ALG-0039**	01/10/2013	CHU Issad Hassani, Beni Messous - Algiers	Potassium dichromate 2.5%	Female	Child	Positive	26.24	Negative	Assemblage A
**ALG-0040**	22/05/2013	CHU Issad Hassani, Beni Messous - Algiers	Potassium dichromate 2.5%	Female	Child	Positive	26.42	Assemblage B	Assemblage B
**ALG-0041**	27/03/2018	Tolga Hospital - Biskra	Potassium dichromate 2.5%	Female	Adult	Negative	26.7	Negative	Assemblage B

**ALG-0042**	27/03/2018	Doctor Saadane Hospital - Biskra	Potassium dichromate 2.5%	Male	Adult	Positive	26.73	Assemblage A	Assemblage A
**ALG-0043**	21/08/2013	CHU Issad Hassani, Beni Messous - Algiers	Potassium dichromate 2.5%	Male	Child	Negative	26.75	Assemblage B	Assemblage B
**ALG-0044**	03/09/2013	CHU Issad Hassani, Beni Messous - Algiers	Potassium dichromate 2.5%	Female	Child	Positive	26.76	Assemblage B + other band(s)	Assemblage B
**ALG-0045**	02/09/2013	CHU Issad Hassani, Beni Messous - Algiers	Potassium dichromate 2.5%	Female	Adult	Positive	27.4	Assemblage B + other band(s)	Assemblage A
**ALG-0046**	27/03/2018	Doctor Saadane Hospital - Biskra	Potassium dichromate 2.5%	Male	Adult	Positive	27.92	Assemblage A	Assemblage A
**ALG-0047**	05/04/2016	CHU Issad Hassani, Beni Messous - Algiers	Potassium dichromate 2.5%	Male	Undetermined	Positive	28	Assemblage B	Assemblage B
**ALG-0048**	27/03/2018	Doctor Saadane Hospital - Biskra	Potassium dichromate 2.5%	Female	Child	Positive	28.36	Assemblage B	Assemblage B
**ALG-0049**	16/09/2013	CHU Issad Hassani, Beni Messous - Algiers	Potassium dichromate 2.5%	Female	Child	Positive	28.41	Negative	Assemblage A
**ALG-0050**	06/10/2013	CHU Issad Hassani, Beni Messous - Algiers	Potassium dichromate 2.5%	Male	Child	Positive	29.5	Negative	Assemblage B
**ALG-0051**	01/08/2016	CHU Issad Hassani, Beni Messous - Algiers	Potassium dichromate 2.5%	Female	Undetermined	Positive	29.76	Negative	Assemblage B
**ALG-0052**	14/04/2013	CHU Issad Hassani, Beni Messous - Algiers	Potassium dichromate 2.5%	Female	Child	Positive	30.03	Negative	Not performed
**ALG-0053**	28/12/2016	CHU Issad Hassani, Beni Messous - Algiers	Potassium dichromate 2.5%	Male	Undetermined	Negative	30.44	Negative	Assemblage A
**ALG-0054**	Undetermined	CHU Nafissa Hamoud, Hussein Dey - Algiers	Ethanol 70%	Male	Undetermined	Positive	30.49	Negative	Not performed
**ALG-0055**	Undetermined	CHU Nafissa Hamoud, Hussein Dey - Algiers	Ethanol 70%	Male	Child	Positive	30.86	Negative	Not performed
**ALG-0056**	14/02/2018	Doctor Saadane Hospital - Biskra	Potassium dichromate 2.5%	Male	Adult	Positive	30.98	Negative	Not performed
**ALG-0057**	10/04/2013	CHU Issad Hassani, Beni Messous - Algiers	Potassium dichromate 2.5%	Female	Child	Positive	31.1	Negative	Not performed
**ALG-0058**	Undetermined	CHU Nafissa Hamoud, Hussein Dey - Algiers	Ethanol 70%	Male	Undetermined	Positive	31.76	Negative	Not performed
**ALG-0059**	Undetermined	CHU Nafissa Hamoud, Hussein Dey - Algiers	Ethanol 70%	Female	Undetermined	Positive	32.18	Negative	Not performed
**ALG-0060**	Undetermined	CHU Nafissa Hamoud, Hussein Dey - Algiers	Ethanol 70%	Male	Undetermined	Positive	32.21	Negative	Not performed
**ALG-0061**	22/09/2013	CHU Issad Hassani, Beni Messous - Algiers	Potassium dichromate 2.5%	Female	Child	Positive	32.23	Other bands	Not performed
**ALG-0062**	08/04/2018	Doctor Saadane Hospital - Biskra	Potassium dichromate 2.5%	Male	Child	Positive	32.27	Negative	Not performed
**ALG-0063**	Undetermined	CHU Nafissa Hamoud, Hussein Dey - Algiers	Ethanol 70%	Female	Undetermined	Negative	33.54	Negative	Not performed
**ALG-0064**	03/10/2018	CHU Nafissa Hamoud, Hussein Dey - Algiers	Ethanol 70%	Female	Adult	Negative	33.84	Negative	Not performed
**ALG-0065**	Undetermined	CHU Nafissa Hamoud, Hussein Dey - Algiers	Ethanol 70%	Male	Undetermined	Positive	34.15	Negative	Not performed
**ALG-0066**	Undetermined	CHU Nafissa Hamoud, Hussein Dey - Algiers	Ethanol 70%	Female	Undetermined	Negative	34.22	Negative	Not performed
**ALG-0067**	15/05/2018	CHU Nafissa Hamoud, Hussein Dey - Algiers	Ethanol 70%	Male	Child	Positive	35.88	Negative	Not performed
**ALG-0068**	26/02/2018	Tolga Hospital - Biskra	Potassium dichromate 2.5%	Female	Adult	Positive	35.93	Negative	Not performed
**ALG-0069**	23/07/2018	CHU Nafissa Hamoud, Hussein Dey - Algiers	Ethanol 70%	Male	Child	Positive	36.68	Negative	Not performed
**ALG-0070**	Undetermined	CHU Nafissa Hamoud, Hussein Dey - Algiers	Ethanol 70%	Undetermined	Undetermined	Positive	36.71	Negative	Not performed
**ALG-0071**	08/10/2018	CHU Nafissa Hamoud, Hussein Dey - Algiers	Ethanol 70%	Male	Child	Positive	36.89	Negative	Not performed
**ALG-0072**	10/10/2018	CHU Nafissa Hamoud, Hussein Dey - Algiers	Ethanol 70%	Male	Child	Negative	37.15	Negative	Not performed
**ALG-0073**	27/05/2013	CHU Issad Hassani, Beni Messous - Algiers	Potassium dichromate 2.5%	Female	Child	Positive	37.43	Negative	Not performed
**ALG-0074**	21/10/2018	CHU Nafissa Hamoud, Hussein Dey - Algiers	Ethanol 70%	Male	Child	Positive	37.47	Negative	Not performed
**ALG-0075**	04/04/2018	Doctor Saadane Hospital - Biskra	Potassium dichromate 2.5%	Female	Child	Negative	38	Negative	Not performed
**ALG-0076**	03/07/2018	CHU Nafissa Hamoud, Hussein Dey - Algiers	Ethanol 70%	Male	Child	Positive	38.39	Negative	Not performed
**ALG-0077**	24/07/2018	CHU Nafissa Hamoud, Hussein Dey - Algiers	Ethanol 70%	Female	Child	Positive	39.1	Negative	Not performed
**ALG-0078**	02/05/2018	CHU Nafissa Hamoud, Hussein Dey - Algiers	Ethanol 70%	Male	Child	Positive	39.93	Negative	Not performed
**ALG-0079**	10/10/2018	CHU Nafissa Hamoud, Hussein Dey - Algiers	Ethanol 70%	Male	Child	Negative	39.97	Negative	Not performed
**ALG-0080**	26/06/2018	CHU Nafissa Hamoud, Hussein Dey - Algiers	Ethanol 70%	Female	Child	Positive	41.51	Negative	Not performed

Table S2. Comparative analysis of the extent of allelic sequence heterozygosity in the *tpi* gene of *Giardia duodenalis* Assemblage B identified in the study.

**Assemblage/Subtype**	**Material**	**Nucleotide position from the beginning of the gene**																				
		**77**	**91***	**105**	**111**	**121**	**132**	**141**	**162**	**165***	**168***	**205**	**216**	**261**	**271**	**280**	**297**	**312**	**333**	**407**	**429**	**448**
**BIII/ 2924 (AY228628)**		C	C	G	G	G	C	C	G	C	C	C	C	G	C	A	A	C	T	A	G	G
**BIV/Ad-19 (AF069560)**		C	T	G	G	G	C	C	G	T	T	C	C	G	C	A	A	C	T	A	A	G
ALG-003	Stool sample	C	C	G	G	G	C	**Y**	**R**	C	**Y**	C	C	G	**Y**	**R**	**R**	C	**Y**	A	G	G
ALG-007	Stool sample	C	T	G	G	G	C	C	G	T	T	C	C	G	C	A	A	C	T	A	A	G
ALG-008	Stool sample	C	C	G	G	G	C	**Y**	**R**	**Y**	**Y**	C	C	G	**Y**	**R**	**R**	C	**Y**	A	G	G
ALG-0017	Stool sample	C	T	G	G	G	C	C	G	T	T	C	C	G	C	A	A	C	T	A	A	G
ALG-0019	Stool sample	C	T	G	G	G	C	C	G	T	T	C	C	G	C	A	A	C	T	A	A	G
ALG-0020	Stool sample	C	**Y**	G	**R**	**R**	C	C	G	C	C	C	C	G	C	A	A	C	T	A	G	G
ALG-0022	Stool sample	**Y**	C	G	G	G	C	C	**R**	**Y**	**Y**	C	C	G	C	A	A	C	T	A	G	G
ALG-0023	Stool sample	C	**Y**	**R**	G	G	C	C	**R**	C	C	C	C	G	C	A	A	**Y**	T	A	G	**R**
ALG-0025	Stool sample	**Y**	C	G	G	G	C	C	**R**	**Y**	**Y**	C	C	G	C	A	A	C	T	A	G	G
ALG-0026	Stool sample	C	T	G	G	G	C	C	G	**Y**	**Y**	C	C	G	C	A	A	C	T	A	G	G
ALG-0030	Stool sample	C	C	G	G	G	C	C	**R**	**Y**	**Y**	C	C	G	C	A	A	C	T	A	G	G
ALG-0031	Stool sample	**Y**	C	G	G	G	C	C	**R**	**Y**	**Y**	C	C	G	C	A	A	C	T	A	G	G
ALG-0032	Stool sample	C	**Y**	G	G	G	C	C	**R**	C	C	**Y**	C	G	C	A	**R**	C	**Y**	A	G	G
ALG-0034	Stool sample	T	C	G	G	G	C	C	A	C	C	C	C	G	C	A	A	C	T	A	G	G
ALG-0035	Stool sample	C	C	G	G	G	C	C	**R**	C	C	C	C	G	C	A	A	C	T	A	G	G
ALG-0036	Stool sample	C	T	G	G	G	C	C	A	C	C	C	C	G	C	A	A	C	T	A	G	G
ALG-0037	Stool sample	**Y**	C	G	G	G	C	C	**R**	C	C	C	C	G	C	A	A	C	T	A	G	G
ALG-0038	Stool sample	C	C	G	G	G	C	C	G	C	C	C	**Y**	G	C	A	A	C	T	A	G	G
ALG-0040	Stool sample	C	T	G	G	G	C	C	G	T	T	C	C	G	C	A	A	C	T	A	A	G
ALG-0041	Stool sample	C	C	G	G	G	**Y**	C	G	C	C	C	C	G	C	A	A	C	T	A	G	G
ALG-0043	Stool sample	C	C	G	G	G	C	C	**R**	C	C	C	C	G	**Y**	**R**	**R**	C	**Y**	A	G	G
ALG-0044	Stool sample	C	C	G	G	G	C	C	**R**	**Y**	C	C	C	G	C	A	A	C	T	**W**	G	G
ALG-0047	Stool sample	C	C	G	G	G	C	C	**R**	C	C	C	C	**R**	C	A	A	**Y**	T	A	G	G
ALG-0048	Stool sample	C	C	G	G	G	C	C	G	**Y**	**Y**	C	C	G	C	A	A	C	T	A	G	G
ALG-0050	Stool sample	C	C	G	G	G	C	C	G	C	C	C	C	G	C	A	A	C	T	A	G	G
ALG-0051	Stool sample	C	T	G	G	G	C	C	G	**Y**	**Y**	C	C	G	C	A	A	C	T	A	**R**	G

Keys: Asterisks (*) represent a substitution patterns suggested as markers for distinct *Giardia* assemblages B subtypes [10,39].

Table S3. Comparative sequence analysis of expressed amino acid substitutions in the *tpi* gene of *Giardia duodenalis* Assemblage B samples identified in the study. Only samples with double peaks resulting in the existence of non-synonymous substitutions were included.

Amino acid sequences	Amino acid position from the beginning of the *tpi* gene							
	26	31	41	87	91	94	136	150
***tpi* reference sequence (GenBank Accession no. KP687784)**	A	Y	V	M	H	I	K	A
ALG-003	A	H	V	M	(H ↔Y)	(I ↔ V)	K	A
ALG-008	A	H	V	M	(H ↔Y)	(I ↔ V)	K	A
ALG-0020	A	(H ↔ Y)	(I ↔ V)	M	H	I	K	A
ALG-0022	(A ↔ V)	H	V	M	H	I	K	A
ALG-0023	A	(H ↔ Y)	V	M	H	I	K	(T ↔ A)
ALG-0025	(A ↔ V)	H	V	M	H	I	K	A
ALG-0031	(A ↔ V)	H	V	M	H	I	K	A
ALG-0032	A	(H ↔ Y)	V	M	H	I	K	A
ALG-0037	(A ↔ V)	H	V	M	H	I	K	A
ALG-0043	A	H	V	M	(H ↔ Y)	(I ↔ V)	K	A
ALG-0044	A	H	V	M	H	I	(K ↔ M)	A
ALG-0047	A	H	V	(I ↔ M)	H	I	K	A

Alignment analysis of the translated nucleotides sequences into amino acid with the complete *tpi* gene reference sequence (GenBank accession no: KP687784.1) indicated that the samples ALG-003, ALG-008, and ALG-0043 had two expressed substitutions of amino acid at positions 91 (H ↔ Y) and 94 (I ↔ V) resulting in replacement of amino acid Histidine to Tyrosine, and Isoleucine to Valine, respectively. Sample ALG-0020 had two expressed substitutions at positions 31 of amino acid (H ↔ Y) and 41 (I ↔ V) inducing replacement of amino acid Histidine to Tyrosine and Isoleucine to Valine, respectively. Samples ALG-0022, ALG-0025, ALG-0031, and ALG-0037 indicated one expressed substitution at position 26 (A ↔ V) triggering a change of amino acid Alanine to Valine. Sample ALG-0023 exhibited two expressed substitutions at positions 31 (H ↔ Y) and 150 (T ↔ A) causing a change of amino acid Histidine to Tyrosine and Threonine to Alanine. Samples ALG-0032, ALG-0044, and ALG-0047 had one expressed substitution at positions 31 (H ↔ Y), 136 (K ↔ M), and 87 (I ↔ M) leading change of amino acid Histidine to Tyrosine, Lysine to Methionine, and Isoleucine to Methionine, respectively. All the replacements of amino acids are reversible.
